# Experiences of LGBT Patients and Families With Hospice and Palliative Care: Perspectives of the Palliative Care Team

**DOI:** 10.1093/geroni/igaa057.218

**Published:** 2020-12-16

**Authors:** Cathy Berkman, Gary Stein

**Affiliations:** 1 Fordham University, New York, New York, United States; 2 Yeshiva University, New York, New York, United States

## Abstract

The lesbian, gay, bisexual, and transgender (LGBT) community experiences discrimination and stigma in accessing health care and social services – including palliative, hospice, and long-term care. Healthcare providers not recognize or address disparities in care. Providers and institutions may be uncomfortable with sexual orientation and gender identity and expression issues, and often don’t inquire about these. LGBT patients fear being open about their identities, not receiving equal or safe treatment, and having their family of choice and designated surrogates disrespected or ignored by healthcare staff. This study examines the degree to which hospice and palliative care providers report inadequate, disrespectful, or abusive care to LGBT patients and family members. A cross-sectional study using an online survey was completed by 865 providers, including social workers, physicians, nurses, and chaplains. Among respondents, 55% reported that LGB patients were more likely than non-LGB patients to experience discrimination at their institution; 24% observed discriminatory care; 65% reported that transgender patients were more likely than non-transgender patients to experience discrimination; 20% observed discrimination to transgender patients; 14% observed the spouse/partner of LGBT patients having their treatment decisions disregarded or minimized; and 13% observed the spouse/partner being treated disrespectfully. Qualitative data are presented to illustrate discomfort with LGBT patients and spouses/partners, disrespectful care, gossip and ridicule, inadequate care, and denial of care. Implications and suggestions for implementing non-discriminatory and respectful institutional and public policy, and for staff education and training to provide competent and respectiful care to this population are presented.

